# Incorporating food addiction into disordered eating: the disordered eating food addiction nutrition guide (DEFANG)

**DOI:** 10.1007/s40519-016-0344-y

**Published:** 2016-12-10

**Authors:** David A. Wiss, Timothy D. Brewerton

**Affiliations:** 1 Nutrition in Recovery LLC, 8549 Wilshire Blvd. #646, Beverly Hills, CA 90211 USA; 2 Psychiatry and Behavioral Sciences, Medical University of South Carolina, Charleston, SC 29425 USA

**Keywords:** Anorexia nervosa, Binge-eating disorder, Bulimia nervosa, Eating disorder, Food addiction, Substance use disorder

## Abstract

Although not formally recognized by the DSM-5, food addiction (FA) has been well described in the scientific literature. FA has emerged as a clinical entity that is recognized within the spectrum of disordered eating, particularly in patients with bulimia nervosa, binge-eating disorder and/or co-occurring addictive disorders and obesity. Integrating the concept of FA into the scope of disordered eating has been challenging for ED treatment professionals, since there is no well-accepted treatment model. The confusion surrounding the implications of FA, as well as the impact of the contemporary Westernized diet, may contribute to poor treatment outcomes. The purpose of this review is twofold. The first is to briefly explore the relationships between EDs and addictions, and the second is to propose a new model of conceptualizing and treating EDs that incorporates recent data on FA. Since treatment for EDs should vary based on individual assessment and diagnosis, the Disordered Eating Food Addiction Nutrition Guide (DEFANG) is presented as a tool for framing treatment goals and helping patients achieve sustainable recovery.

## Introduction

The classic prototype of a person with an eating disorder (ED) is a middle class young white female with anorexia nervosa (AN). However, recent prevalence data indicate that EDs are common in both genders, occur at all ages, in all races, and affect all income levels [1]. In the past decade, EDs have been reported across wider demographics due to factors such as increased awareness, changing societal values, targeted research, and an evolving Western diet. With the sharp rise in substance use disorders (SUDs), mental health professionals are seeing increased numbers of patients with both SUD and ED. While the interaction between SUDs and EDs is not fully understood, there are bidirectional associations in research conducted on females with AN and bulimia nervosa (BN) [2, 3]. Evidence linking binge-eating disorder (BED) to addiction (alcohol, drug, food) has strengthened in recent years, although considerable controversy remains about the nature of this association. In a recent textbook, Brewerton and Dennis [4] explored links and correlations between EDs, SUDs, and addictions across genetic, neurobiological, and behavioral domains, and advocated for an integrated treatment approach. Frank [5] suggests that a “dimensional approach that identifies neurobiological underpinnings of psychiatric disease based on specific behavioral constructs may help eventually develop more accurate models of EDs and identify empirically more specific and effective biological treatments”.

Although not formally recognized by the DSM-5, food addiction (FA) has been well described in the ED and obesity literature, and a recent meta-analysis reported that 20% of all subjects (60% were female and overweight/obese) tested for FA met criteria based on the Yale Food Addiction Scale (YFAS) [6]. Incorporating the concept of FA into the spectrum of disordered eating has been difficult for ED treatment professionals for a variety of reasons, including training suggesting that EDs are unique disorders distinct from addictions, the dogmatic belief that there are “no bad foods” (everything in moderation), and because there is no well-established treatment for FA. Several authors have recommended the application of traditional addiction treatment [7] including psychiatric interventions [8] as well as reduced exposure to addictive foods [9, 10]. An alternative approach advocates reducing exposure to restrictive eating (dieting) since it may perpetuate the cycle of disordered eating [11, 12]. Many ED professionals are uneasy conceptualizing EDs as a form of addiction, since the classic eating disorder AN restricting type (AN-R) does not resemble an addictive disorder (although it can be argued that “starvation” can become addictive) [13, 14]. Unfortunately, standard ED treatment is associated with high rates of relapse and poor long-term remission rates [15]. Some authors believe that existing treatments fail because they do not address impulsivity as a core factor of eating pathology [16]. The confusion surrounding the concept of FA, as well as the impact of the contemporary Westernized diet, may contribute to poor treatment outcomes. The purpose of this paper is twofold. The first is to explore the relationships between FA, SUDs and EDs, and the second to propose a new model of conceptualizing and treating EDs that incorporates recent data on addiction.

**Fig. 1:**
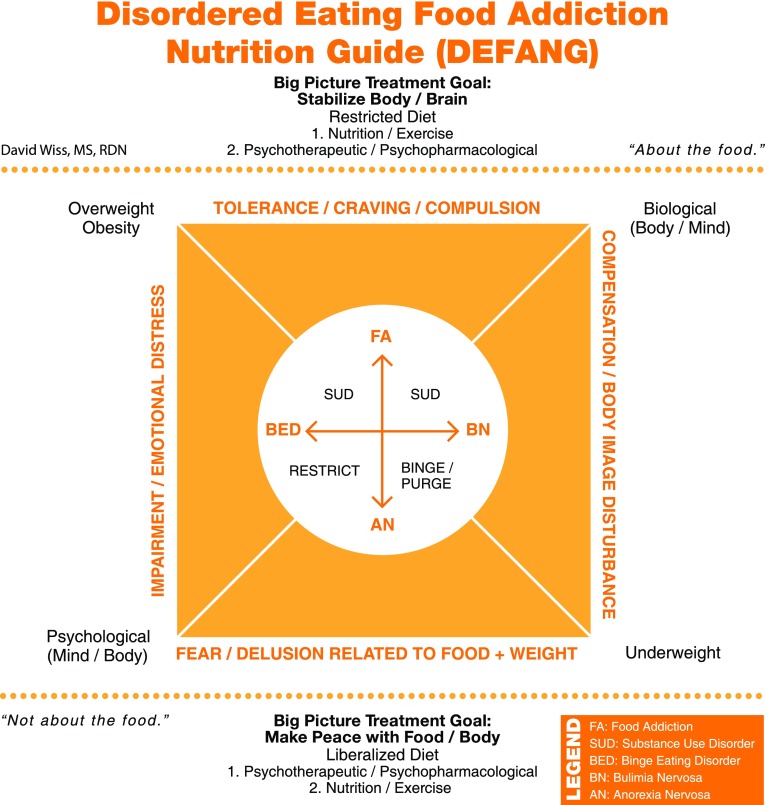
A conceptual framework for individualized nutrition interventions designed to promote sustainable eating disorder recovery

The Disordered Eating Food Addiction Nutrition Guide (DEFANG) was developed for clinical application at treatment facilities for FA, SUDs, EDs, and related disorders, such as post-traumatic stress disorder (PTSD). The objective is to plot patient symptoms onto a diagram (outside of the circle, inside of the square) in order to craft effective, individualized intervention strategies (see Fig. 1). Since classic ED treatment does not typically acknowledge the gravity of addictive processes (including food), patients with addictions report struggling with messages that are inconsistent with their experience (e.g., “eat all foods in moderation”). The generic message of “moderation” is subjective (perceived), remains poorly defined, contains self-serving biases, and appears to reduce self-conflict around over-consumption of food [17]. While reduction of self-conflict (e.g., shame) is critical for ED recovery and the development of a sustainable relationship to food, the misinterpretation and misapplication of “moderation” can become counter-productive, particularly when there is addictive symptomatology. The DEFANG is designed to minimize inconsistent messaging from the treatment team by necessitating that the psychiatrist, therapists, dietitians, and supporting staff reach an agreement about the patient’s diagnosis and “big picture” treatment goals. Multidisciplinary staff can plot patients on the DEFANG based on their own assessment and then reach a consensus. Additionally, patients can be educated on the use of the DEFANG and invite them to plot themselves on the diagram, which enables self-assessment to be part of their treatment plan. Patients should be instructed that not all ED cases require the same treatment approach, which may decrease comparisons that can plague an ED unit.

## Anorexia nervosa (AN)

The DSM-5 diagnostic criteria for AN include an intense fear of gaining weight or becoming fat, and persistent behavior that interferes with weight gain, even at a significantly low weight [18]. Appearance concerns that are not weight related (e.g., skin, nose, body hair, teeth) have also been reported in individuals with AN. Among females with AN, the restricting type is less likely to have an SUD than the binge-eating/purging-type [19], which may be due to higher rates of impulsivity associated with bulimic symptomatology [20]. Impulsivity is less common in AN than in other EDs and may be a predictor of those with AN-R who cross over into binge/purge behavior (AN-BP) [21]. Thus, diagnostic crossover between different EDs is predictable, particularly with AN-BP, BN, and BED. Women with AN may engage in substances initially in an effort to lose weight, whereas women with BN may turn to drugs or alcohol to subdue bulimic urges [2]. The caloric contribution of alcoholic beverages may deter patients with AN from excessive use, although controlled and calculated consumption is not uncommon. Of all the EDs, AN-R is the least compatible with SUDs since individuals with AN-R maintain the ability to consistently abstain, are less likely to exhibit cravings, and may not experience impaired behavioral control [13]. While it can be argued that AN-R may be a form of starvation dependence or dieting addiction, this is yet to be established. Thus, AN sits on the opposite spectrum of FA (see Fig. 1), and for many of these patients the disorder is less “about the food” and more related to underlying psychological factors, i.e., irrational fears, distorted perceptions and beliefs (likely unrelated to food addiction), which varies from patient to patient. However, FA symptomatology has been reported in patients with AN-BP [22], further establishing the need to look at these disorders on a continuum.

## Bulimia nervosa (BN)

Individuals with BN have an altered sense of self-evaluation which is unduly influenced by body shape and weight [18], and share the characteristic body image disturbance with AN. While similarities between AN-R and BN exist, there are clear differences such as the ability to consistently control eating, whereas AN-BP and BN more closely resemble one another. Patients with BN compensate for their loss of control around food through various forms of purging, fasting, or excessive exercise, usually in an effort to prevent weight gain. BN results in altered reward sensitivity in the dopaminergic brain system, which may increase addictive potential [23]. Muele [24] speculates that many bulimics who are in a normal weight range may be food addicts. Associations between BN and alcohol use disorders (AUDs) have been linked to common genetic influences [25]. Similarly, overlap between BN and SUDs has been attributed to genetic more than environmental factors [26]. Alcohol consumption that reduces food cravings in BN may be due to increased satiety, reduced attention to eating-related urges, or simply by improving mood [27]. Nonmedical prescription stimulant abuse used for appetite suppression and weight loss has been associated ED symptomatology including bingeing and purging [28]. Thus, SUD (including alcohol) is identified as a characteristic overlapping with BN (see Fig. 1).

It remains unclear if the addictive symptomatology in BN patients is more closely associated with the eating or the compensatory behaviors [29]. The DSM-5 now recognizes a purging disorder, which occurs in the absence of binge eating, suggesting that purging by itself can be a rewarding experience (also evidenced by AN-P). The apparent overlap between BN and FA suggests that reducing exposure to addictive foods may be effective in the treatment of BN [29]. Research has shown that the top fifteen foods considered to be most addictive by individuals with FA include chocolate, ice cream, French fries, pizza, cookies, chips, cake, popcorn, cheeseburgers, muffins, breakfast cereal, gummy candy, fried chicken, sugary soda, and rolls [30]. All these highly palatable foods are processed foods and are noted to share pharmacokinetic properties with drugs of abuse, such as concentrated dose and rapid rate of absorption. However, the traditional nutritional approach to BN has been to liberalize the diet, given that food restriction may increase reward sensitivity [31] and promote rebound bingeing. Umberg et al. [23] proposed that BN may be separated into two distinct sub-types that recognize patients who are hyporesponsive to reward (akin to AN) versus those with hypersensitive reward circuitry (akin to FA). This distinction separates those with BN into two groups that would warrant different treatment approaches (Fig. 1).

## Binge-eating disorder (BED)

Important diagnostic components of BED include impairment and distress [18], which may come in the form of anxiety [32]. Body dissatisfaction, shame, severe self-criticism, social comparison, stress, and trauma are common in BED, as well as in BN and AN (especially AN–BP subtype), and have been identified as underlying mechanisms behind binge behaviors [33–36]. Consumption of highly processed palatable foods with motives to reduce posttraumatic symptoms, such as hyperarousal, intrusive images, and facilitate numbing has been associated with BED, BN and bulimic behaviors in general [33, 37, 38]. Obese individuals with BED report a lower quality of life than if either of those conditions exists alone [39]. While maladaptive emotional regulation is a hallmark of BED, weight or BMI is not considered criteria for diagnosis. However, when there is BED and FA, obesity is likely to occur [40]. For individuals who have successfully lost weight, binge-eating behavior is the primary mechanism behind weight regain [41]. Thus, BED treatment may interrupt a steep weight gain trajectory [42].

Currently, the most common nutritional treatment for BED appears to resemble the treatment for AN-R, where patients are encouraged to liberalize their diet and learn to consume all foods, including highly palatable foods, in moderation. This treatment strategy might be effective for a BED patient whose eating pathology stems from psychologically based (e.g., shame) rather than biologically based (chronic exposure to highly palatable foods) mechanisms, but may be less effective over a longer period of time if the patient is FA primary. In other words, therapeutic interventions alone will not treat BED/FA if the patient continues to eat addictively (clinical anecdote). Approximately, one-fourth (27%) of BED patients have a co-occurring SUD [43]. Shared mechanisms in addictive disorders and BED include: reward dysfunction, craving, emotion dysregulation, and impulsivity [44]. Recent findings by Aloi et al. [45] suggest neurocognitive differences between patients with AN (rigidity and attention to details) compared to BED (lack of attention and difficulty adapting to changes), which point to the need for different intervention strategies. Meanwhile, many BED patients exhibit food fears and restrictive eating patterns similar to AN patients, hence the overlap shown in Fig. 1. It has been suggested that BED be treated in a way that acknowledges the presence of a range of binge-eating phenotypes [46] and subtypes, including co-occurring SUD [43].

## Food addiction (FA)

Food addiction has been described as a reward-responsive phenotype of obesity [47] although FA can exist without obesity [48] and without BED. Gearhardt et al. [40] reported that approximately half of BED patients meet criteria for FA utilizing the YFAS, suggesting the presence of a more disturbed subset than BED or FA alone. Among ED patients, probability of FA is predicted by high negative urgency, high reward dependence, and lack of premeditation [49]. Not surprisingly, there is evidence that FA can undermine efforts to lose weight related to behavioral (e.g., tolerance and withdrawal) and neurophysiological (dopamine and endogenous opioid action in the midbrain) pathology [50]. Alterations in dopamine neurocircuitry observed in obese individuals can make food less rewarding and more habitual [51]. Rates of weight regain in weight-reduced obese individuals are reminiscent of the high relapse rates for addiction [52]. Highly processed foods (refined grains, added sugars, sweeteners, fats, and salt) that share characteristics of drugs of abuse (e.g., high dose, rapid rate of absorption) are most associated with food addiction [30]. These authors found the combination of white flour, sugar, and fat (e.g., cookie) to be the most addictive combination. Given the abundance and easy access of highly palatable food, craving and compulsion are an important construct to consider within the biological context of EDs [8].

Dysfunction of the dopamine D2 receptor leading to substance-seeking behavior (alcohol, drug, food) has been termed reward deficiency syndrome (RDS), a concept that unites addictive, impulsive, and compulsive behaviors [53]. RDS remains somewhat controversial as causal in addiction and obesity. A recent review identified behavioral changes related to the A1 allele (associated with RDS) including novelty seeking, delay discounting, impulsivity, and ability to avoid negative consequences as key mechanisms behind hyperphagia [54]. Low availability of dopamine D2 receptors in the nucleus accumbens reduces activity in the prefrontal cortex, contributing to impulsivity and poor self-control in obese individuals [55]. This reinforcement pathology favors unhealthy behaviors that contribute to weight gain [56]. Alternatively, reward surfeit theory suggests that individuals with greater reward region sensitivity (rather than less) to substance-related cues are at elevated risk for overeating, suggesting that habitual intake of palatable foods leads to hyper-responsivity of attention and reward valuation [57].

While impaired dopaminergic signaling mechanisms have been implicated in individuals with FA [58], recent investigators have explored hormonal differences between obese individuals who meet criteria for FA versus those who do not [59]. The arcuate nucleus in the hypothalamus is the integration site for blood-borne signals (e.g., hormones, nutrients) that calibrate the brain reward system governing hedonic feeding [60]. Several authors have identified leptin as the indirect link between overeating and addiction, since it has action extending to the brain reward circuits influencing preference for highly palatable foods. Leptin-deficient individuals could be classified as meeting criteria for FA [61]. There is also evidence that the insulin receptor signaling pathway interferes with leptin signaling, suggesting that hyperinsulinemia may contribute to leptin resistance [62]. Other reports implicate that the ghrelin system (gut-derived) may alter the set point of the dopaminergic neurons in the ventral tegmental area, thereby enhancing the ability of rewarding substances to activate the midbrain dopamine system [63, 64].

While it is outside the scope of this review to discuss the various mechanisms of neurotransmitters and hormones associated with disordered eating, emerging data should direct new specific biological strategies when treating patients with addictive symptoms. Recently, the gut microbiome has received increased attention as a contributing factor to obesity [65, 66] and EDs [67]. The gut-brain axis involving receptors in the enteric nervous system, the autonomic nervous system (including vagus nerve), immune system, neuroendocrine system, systemic circulation (including lymphatic), and the spinal cord implies a “cross-talk” mechanism between gut microbiota and the host brain [60, 68]. Fermentation byproducts of fiber degradation by microorganisms include short chain fatty acids (SCFAs) that are involved in the synthesis of hormonal precursors, suggesting that SCFAs modulate the enteric nervous system [69]. New insights suggest that the composition of food consumed (e.g., fiber content, artificial sweeteners) impacts the microflora, which in turn regulates homeostasic mechanisms [70–72]. It is imperative that we look beyond calories (quantity) towards the impact of food (quality) on gut health when tailoring nutrition interventions for disordered eating, particularly when hedonic mechanisms appear to override homeostatic mechanisms.

The general public appears to support obesity treatment through the lens of addiction [73], but believe that obese individuals are personally responsible for their weight. The food industry continues to deny responsibility for creating obesogenic environments by continuing to stress individual responsibility for eating, and continuing to emphasize moderation. Put in perspective, educational efforts alone have not reduced the use of illicit drugs. Recently, it has been shown that belief in the addictive potential of a substance or behavior creates support for policies intended to curb their use [74]. Efforts to influence the food industry (“food environment”) may be required to combat the rising epidemic of FA, obesity, and many cases of BN and BED. Psychological interventions (traditionally considered first-line in the treatment of EDs) alone are not sufficient in FA. There is a clear need to treat the gut, brain, and endocrine system together for recovery efforts to be sustainable and effective. For many patients with FA, it is “about the food” in addition to various underlying factors; therefore, highly specified nutrition (and exercise) interventions should be considered a primary part of a treatment program [75].

## Substance use disorder (SUD)

Patients with SUDs share characteristics with compulsive overeaters from brain imaging studies [10] and behavioral models [76]. Individuals in early addiction recovery have described dysfunctional eating such as bingeing to satisfy drug cravings [77], leading to unhealthy and undesirable weight gain [78]. Altered fat regulation observed in cocaine-dependent men has been linked to lower levels of circulating leptin [79], lending support to the significance of hormones relative to addictive processes. Evidence that gastric bypass patients develop new-onset SUDs/AUDs in the post-surgical period (second year or later) suggests that “addiction transfer” is a likely phenomenon that may require specialized treatment [80–82]. Interestingly, there is evidence of reduced appeal of high-energy foods in patients after gastric bypass [83] suggesting changes in brain reward that may be linked to alterations in gut microbiota. Several diagnostic characterisitics of SUD overlap with BED, such as using larger amounts than intended, continuing to consume despite negative consequences, and reducing other pleasurable activities due to consumption. Currently there is a need for empirically supported treatments for co-occurring SUD and ED [84]. ED symptoms have been significantly associated with treatment rejection among young adult men in residential SUD treatment, suggesting that comorbid ED and SUD are a higher risk clinical group [85]. Thus, SUD is associated with BN, BED, and FA in Fig. 1.

## The disordered eating food addiction nutrition guide (DEFANG)

The bottom half of the DEFANG describes classic ED treatment that is seemingly geared toward the underweight patient with AN where it is often times “not about the food”. This is not to suggest that weight restoration via nutrition therapy is not of paramount importance in the treatment of AN, but rather it is not the primary focus in the larger scheme (after re-feeding and stabilization). In many cases, the AN patient will insist that it is “about the food” and thus be resistant to a meal plan. The big picture treatment goals are to make peace with food by overcoming dietary fears and unnecessary restrictions (liberalize the diet, apply moderation), make peace with body by engaging in therapy addressing body image, and in many cases psychotropic medication to stabilize mood and to lower obsessional anxiety. Psychotherapeutic interventions are considered the “big picture” foundation of treatment, with nutrition and physical wellness (e.g., exercise) being secondary. In other words, nutrition and exercise interventions alone are not sufficient treatment for AN. If the patient does not begin to make some peace with food and body, recovery is not likely to be sustainable.

The upper half of the DEFANG proposes treatment strategies for the overweight/obese patient with FA, displaying clear signs of addiction (e.g., tolerance and craving) and compulsion around food intake. These cases are more likely to be “about the food”, thus containing biological underpinnings that must be addressed in treatment. Treatment goals are to stabilize the body (e.g., gut, hormones) and begin to heal the dysfunctional brain circuitry that persists in addiction. This will require a restricted diet with reduced exposure to highly palatable foods and caloric upper limits. “Restricted” does not mean eliminating food groups or utilizing low calorie diets, but rather restricting the excessive and undesirable consumption that is characteristic of substance-related addictive disorders. Nutrition and exercise interventions should thus be considered key components. Therapy and/or medication may be important in the treatment of FA, but it is insufficient to address the physical ramifications associated with substance abuse alone. However, therapeutic interventions that are designed to support the journey towards stabilization of the body and brain can prevent the patient from fostering disordered thinking, such as placing too much emphasis on weight or developing irrational food fears, and will be critical for sustainable recovery.

Assessment and plotting of patients with BN and BED using the DEFANG may prove to be helpful for case conceptualization. If a patient displays classic ED psychopathology (akin to AN), they will be placed in the bottom half of the model, whereas a client displaying addictive symptomatology will be plotted in the upper half. The upper left corner represents the obese food addict with BED, whereas the bottom right corner represents the underweight restrictive eater (AN or BN). Movement towards either of these corners reflects weight/BMI status, which will be useful in painting a more complete picture. It can also be useful to specify severity as outlined in the DSM-5 (mild, moderate, severe, extreme). If a patient is plotted near the mid-section of the diagram, they will benefit from a combination of treatment approaches, which should be determined by the clinical staff. For example, a BED patient in the upper half (but near the middle) of the DEFANG may benefit from having dessert only once or twice per week whereas a BED patient in the lower half may require dessert more often. Furthermore, a patient with BN on the lower half of the model may be better suited in a milieu with anorexics (liberalized diet), whereas a BN patient in the upper half may benefit from targeted education about the potential impact of food processing on eating behavior. A middle-ground message can be: “All foods fit. But not all foods fit for all people. And just because the food industry manufactures and sells it, does not mean we have to include it”.

## Case study

### History

A 46-year-old, 180.5 kg (BMI = 59), woman with a history of depression entered residential treatment for BED in June 2015. While the patient had never been to a treatment center before, she had been treated for depression and had seen a therapist intermittently for the past 14 years, mostly at age 32 (2001).

She described both of her parents as well as her grandparents being alcoholics. She reported that her mother was “skinny”, but her father battled with his weight his entire life. The patient was the victim of sexual trauma at age 9 but was never diagnosed with PTSD, or formally treated for it (aside from trauma-informed group therapy utilizing psychodrama techniques during treatment). At age 11, she learned how to count calories and exercise with her parents. The patient stated that she had been on many diets in her lifetime, including Jenny Craig, Weight Watchers, and Atkins [86]. She began binge drinking in high school and described herself as a “blackout drunk”. During her twenties, she was a heavy social drinker and often drank to excess. At this time, she also used cocaine, marijuana, and crystal meth briefly. She reported that food had always been her primary addiction but stated “If I’m eating, I’m usually drinking” preferring starchy/fatty/salty foods to sweet foods, with her favorites being cheeseburger, pizza, pasta, French fries, bread, and ice cream. The patient states that she eats large amounts of food all day long, consistently seeking a “full” feeling. She does not endorse restrictive eating patterns that are common with BED patients, although she will wait until she has privacy and “complete comfort” to eat her favorite foods.

The patient’s weight reached approximately 136 kg in 2005. She utilized the 12-Step program Compulsive Eaters Anonymous–Honesty, Open-mindedness, Willingness (CEA-HOW) and lost 68 kg by adhering to a rigid food plan, attending meetings, and working the Steps with a sponsor. The patient described feeling that for the first time in her life that she was “not a slave” to food, being a “different person” and experiencing internal freedom. She also stopped drinking alcohol and using drugs at that time and maintained her sobriety and weight at 68 kg for 6 months until she became pregnant. Unfortunately, the patient moved to another area where there was no CEA-HOW meetings, and stopped actively working her recovery program. After being separated from her recovery program, the patient distinctly recalls eating a Reuben sandwich and then “decided” to reincorporate bread back into her diet, which led to rapid weight gain. Although she tried to revert back to her CEA-HOW food plan, she was unsuccessful and could not “put 30 days together.”

The patient got married in 2007, but gained 45 kg shortly after the wedding. She divorced in 2010 and struggled with her weight as a single mom (ranging from 136 to 181 kg), reporting that her son also struggled with food and weight issues. The patient did not seek psychotherapy at this time despite the fact that she reported being very challenged by the stressors of life as a single mother. In 2012, the patient found CEA-HOW meetings online and utilized Alcoholics Anonymous (AA) to get sober and lose 45 kg. Sadly, as a single mom, the patient could not stay connected to the program and maintain her recovery.

### Treatment

**Fig. 2:**
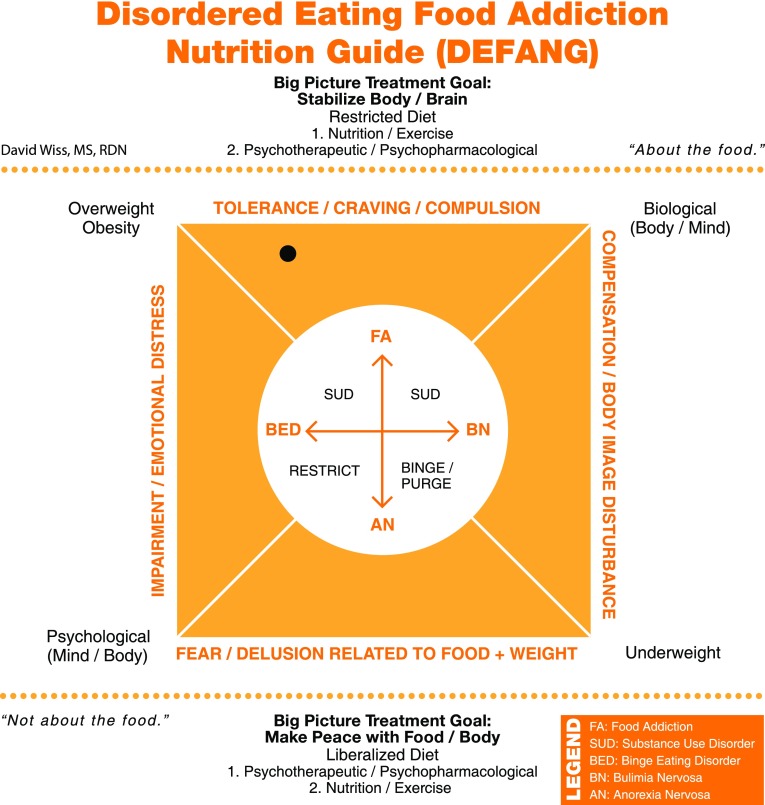
Patient displays symptomatology that resembles FA (using criteria for SUD) more clearly than BED. Thus, the patient is plotted near the uppermost portion of the DEFANG but approximately halfway towards the BED domain. Relative orientation was agreed upon by the patient and clinical team to individualize nutritional treatment and education. Other patients in treatment who fell into other domains of the DEFANG received different food and messaging that was determined more appropriate for their ED diagnosis

The patient entered residential treatment for BED in June 2015 and remained there for 4 months. The patient was started on citalopram 40 mg for depression, which was helpful. She was convinced that she needed a structured abstinence-based approach towards eating, stating that throughout her entire life she had never been able to eat in “moderation”. After a comprehensive intake and assessment by Registered Dietitian Nutritionist (RDN) and use of the YFAS combined with the DEFANG, it was determined that she belonged to the uppermost portion of the model, towards the upper left corner (see Fig. 2). The patient and RDN agreed on a nutritional treatment plan that involved no dessert and no exposure to highly palatable foods. She understood that she was receiving treatment for her food addiction, affirming that she only came to treatment because she knew it would be addressed as an addiction, rather than a classic eating disorder. The patient was resistant to the message of “body acceptance” and “body positivity” presented to her during treatment.

The patient was put on a meal plan that included 3 meals and 3 snacks (2400–2600 kcal), with several options for breakfast and snacks. Her meal plan included representation from all food groups several times/day that was high in dietary fiber. The patient did not consume highly palatable foods in treatment, specifically no fried foods, white flour, or foods with added sugar. The patient eagerly attended weekly OA meetings during treatment and connected with a sponsor. She exercised with a trainer 9×/week, with 2 workouts 3 days/week and one rest day. The patient was weighed “blind” during treatment, with the plan to discuss her weight at the halfway point (60 days) and at discharge. At day 60, the patient had lost 13.6 kg but was very upset with her modest weight loss. This patient spent a large portion of her life equating her weight with her self-worth and had a difficult time acknowledging her mental, emotional, and spiritual recovery. The patient had previously lost 45+ kg on a CEA-HOW (low calorie) meal plan (3 meals, no snacks) on two separate occasions. She was tearful and threatened to leave treatment but was convinced to stay by the clinical staff. Her treating RDN explained that low calorie meal plans result in rebound bingeing and eventually lead to rebound weight gain, and that she was put on a meal plan and calorie level that she could more realistically sustain over time. In conjunction with her primary therapist, her meal plan was reduced to 3 meals, 2 snacks (2000–2200 kcal). She also engaged in cooking classes during treatment and was taught to “plate” her own food and eyeball her portions. The patient was discharged with this meal plan (3 meals, 2 snacks) and was instructed not to “diet” (count calories, weigh and measure food, etc.). She discharged weighing 146.5 kg.

### Follow-up

The patient followed her discharge meal plan and did not eat foods determined to be addictive (fried foods, white flour, and added sugars). She got a job at a gym as a membership representative and exercised on most days. The patient became involved in Overeaters Anonymous (OA), began working with a sponsor who kept her accountable and she gradually lost another 22.7 kg (down to 123.8 kg) while continuing outpatient psychotherapy. Eventually, in an attempt to reduce “black and white thinking”, the therapist recommended that she should eat in “moderation”. At first resistant, she recalls: “the addict in me thought this was a really good idea”. The patient then had a “slip” on sugar while losing the motivation to exercise regularly. She gained 13.6 kg in only 2 months while struggling to “get back” due to general life stress. At recent follow-up, the patient reported weighing 138 kg and continued to struggle with her food addiction, although most recently she was re-engaged with CEA-HOW phone bridge meetings and had a renewed sense of optimism.

## Conclusion

The treatment for EDs should vary based on the individualized assessment and diagnosis. All patients entering ED treatment should not receive the same type of food and generic nutrition education. Using the DEFANG, treatment teams can provide consistent ED-informed messages that are consistent with the patient diagnosis, and that can challenge self-destructive “black-and-white thinking”. Meanwhile, educational components can acknowledge the profound impact that contemporary food has on the human gut, brain, and endocrine system. Providers should explore these concepts with their patients and be willing to validate their difficulties navigating the food supply. Food addiction is a valid construct that should be incorporated into the spectrum of disordered eating, particularly for those patients with BN, BED, and co-occurring addictive disorders (FA or SUD). Further research on brain structure and function will help better model the complex interaction between EDs, SUDs, and addictions. Currently, there is no consensus on how to most effectively treat FA; therefore, efforts to identify more specific nutrition intervention strategies are clearly needed.
